# *Linguatula serrata* Tongue Worm in Human Eye, Austria

**DOI:** 10.3201/eid1705.100790

**Published:** 2011-05

**Authors:** Martina Koehsler, Julia Walochnik, Michael Georgopoulos, Christian Pruente, Wolfgang Boeckeler, Herbert Auer, Talin Barisani-Asenbauer

**Affiliations:** Author affiliations: Medical University of Vienna, Vienna, Austria (M. Koehsler, J. Walochnik, M. Georgopoulos, C. Pruente, H. Auer, T. Barisani-Asenbauer);; University of Kiel, Kiel, Germany (W. Boeckeler)

**Keywords:** *Linguatula*, Pentastomida, anterior chamber, uveitis, 18S ribosomal DNA, tongue worm, parasites, molecular data, Austria, dispatch

## Abstract

*Linguatula serrata*, the so-called tongue worm, is a worm-like, bloodsucking parasite belonging to the Pentastomida group. Infections with *L. serrata* tongue worms are rare in Europe. We describe a case of ocular linguatulosis in central Europe and provide molecular data on *L. serrata* tongue worms*.*

The species *Linguatula serrata* belongs to the Pentastomida, a still-enigmatic group of worm-like, bloodsucking parasites that inhabit the upper respiratory tract of terrestrial, carnivorous vertebrates, mostly reptiles and birds; *L. serrata*, commonly called tongue worms, typically inhabit canids and felids. The intermediate hosts of these parasites are usually sheep, cattle, or rodents. The hosts ingest the eggs, and the first instar larva hatches within their intestines, penetrates the mucosa, and retreats into the tissue, where it encysts and molts to the third larval stage. Humans can serve as aberrant final hosts after ingesting raw or poorly cooked viscera (i.e., liver, lungs, and trachea) of intermediate hosts. This nasopharyngeal infection is known as Halzoun syndrome in the Middle East or as Marrara in Sudan (*1**,**2*). Humans can also serve as accidental intermediate hosts, when ingesting the eggs (visceral infection) (*3*). Intraocular infection is extremely rare; only 5 cases caused by *L. serrata* tongue worms have been described: 2 from the southern United States (*4**–**5*), 1 from Portugal (*6*), 1 from Israel (*7*), and 1 from Ecuador (*8*).

The phylogenetic position of the pentastomids is still not fully resolved. A position as the sister group of branchiurian crustaceans (Maxillopoda) is supported by molecular data and sperm morphology (*9**–**11*) and is widely accepted today; however, sound evidence also exists for other classifications (*12*). The group itself is divided into 2 orders, the more primitive Cephalobaenida and the more advanced Porocephalida. All species infecting humans are currently classified as Porocephalida; the species *L. serrata* and *Armillifer armillatus* are responsible for most human cases of infection.

## The Study

A 14-year-old girl was referred to the eye clinic at the Medical University of Vienna with an unknown parasite detected during ophthalmologic examination. The girl had redness, pain, and progressive visual loss in the right eye. Her medical history was unremarkable except that she had reported regular contact with domestic animals: 2 dogs, cats, and 1 turtle. She had no history of bites or other infestations, and neither she nor any of her animals had been abroad.

Vision was reduced to 0.1 Snellen. Slit lamp examination revealed a mobile parasite swimming like a fish in the anterior chamber of the eye (Video) signs of local inflammation with cells and Tyndall phenomena were present. The pupil was round and reactive, the lens was clear, and a slight iridodonesis was observed. Fundus examination through a constricted pupil showed no abnormalities. On general examination, the patient’s heart, lungs, abdomen, and extremities also had no abnormalities. Her nose and throat were examined carefully for additional parasites. Serologic data including serum chemistry, C-reactive protein, fasting blood glucose, erythrocyte sedimentation rate, creatinine, and blood urea nitrogen levels were within normal limits. Her complete blood count revealed cell differentiation within normal limits without eosinophilia. Her chest and sinus radiographs showed no abnormalities. Surgical removal of the parasite was complicated because of high mobility of the parasite inside the anterior chamber. The worm escaped into the posterior segment of the eye where it was found, after lens removal and complete vitrectomy, in a recess of the ciliary body. A viable parasite was extracted and transferred to physiologic saline. One month later, the eye was completely free of irritation, and 3 months later an artificial intraocular lens (ARTISAN; OPHTEC BV, Groningen, the Netherlands) was implanted. Final visual acuity was 1.0 Snellen.

**Video:**
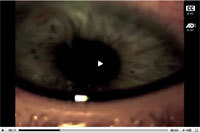
*Linguatula serrata* tongue worm swimming in an infected human eye.

Direct Link: http://streaming.cdc.gov/vod.php?id=206de2efe9617387b685230c158245ba20110426172459732

The surgically extracted parasite was examined microscopically and then subjected to DNA isolation with the QIAGEN tissue kit (QIAGEN, Hilden, Germany). The 18S rDNA was amplified and sequenced stepwise by using 6 internal primers (P1fw, P1rev, P2fw, P2rev, P3fw, P3rev) (*13*) and a 310 ABI PRISM automated sequencer (Applied Biosystems, Darmstadt, Germany); 3 sequences each were obtained from both strands. Fragments were combined to a consensus sequence with the GENEDOC sequence editor (www.nrbsc.org/gfx/genedoc), and deposited in GenBank (accession no. FJ528908). For cluster analysis, primer sites, unique gaps, and ambiguously aligned sites were excluded, resulting in a dataset of ≈1,560 aligned sites. Cluster analysis was performed by using the PHYLIP 3.63 package (www.phylip.com), with neighbor joining, maximum parsimony, and maximum likelihood as evolutionary models and 100 bootstrap replicates generated for each model. Trees were rooted with 4 branchiurian sequences (GenBank nos. DQ925818, DQ925819, AF436004, and DQ925842).

**Figure 1:**
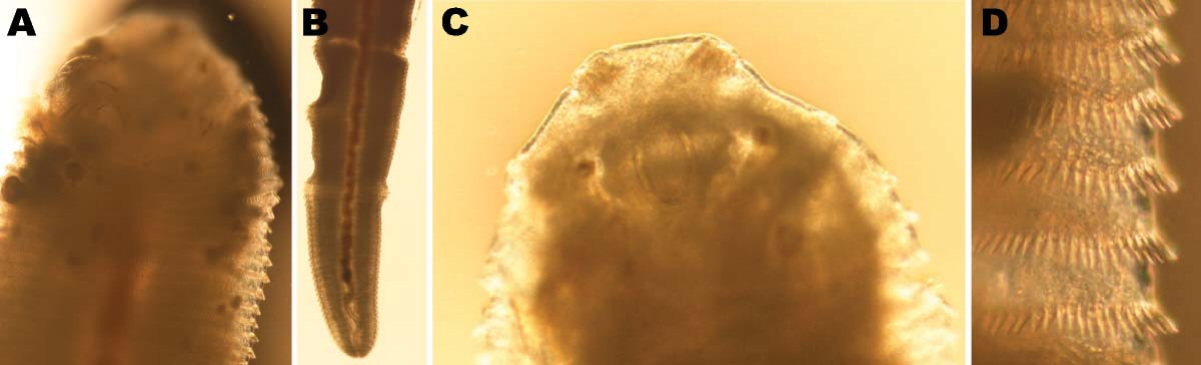
Morphology of *Linguatula serrata* tongue worm. A) Ventral anterior end with hooks. B) Posterior end laterally with primordial uterus, genital porus, and intestine (note peristalsis). C) Dorsal anterior end with cuticular structures of apical papillae, chitinoid oral clasp, and insertions of oral muscles. D) Rows of spicules. Original magnification ×50 (A–C) or ×100 (D).

The parasite was 4.5 mm long, appeared whitish, and had a flattened (tongue-like) body shape with a rounded anterior (0.9 mm wide) and pointed posterior end (0.35 mm). It had curved hooks with sharp tips on the anterior ventral side (Figure 1, panel A), a red primordial uterus stretching from the anterior to the posterior end, a median ventral genital porus, and a terminal anus (Figure 1, panel B). The anterior end had apical papillae and a chitinoid oral clasp (Figure 1, panel C). The cuticle showed 94 rings and conspicuous spicules (Figure 1, panel D). Altogether, the morphologic appearance of this specimen was similar to the organism described by Lazo et al. (*8*) and could thus be identified as a third instar larva of *L. serrata* tongue worm. The cuticular rings and the spicules at the posterior margins of the cuticle rings are also characteristic for this parasite (*14*).

We assumed the mode of infection to be ingestion or direct eye contact with *L. serrata* eggs and the source of infection to be 1 of the patient’s 2 pet dogs. Dogs are the typical final hosts of *L. serrata* tongue worms, and children generally tend to have carefree contact with pet animals. Unfortunately, neither dog could be investigated for potential infection. Infection rates of dogs in Austria are unknown; only 1 case has been reported, and it occurred in a dog imported from the United States (*15*). The girl also had cats and a turtle, but cats are not primary hosts and turtles are never hosts for the life cycle of the *L. serrata* tongue worm (*14*). Two of the other 5 ocular linguatulosis case-patients mentioned earlier had a recent history of ocular trauma but whether trauma was related to infection remains unknown. In the case reported here, no eye injury was noted.

**Figure 2:**
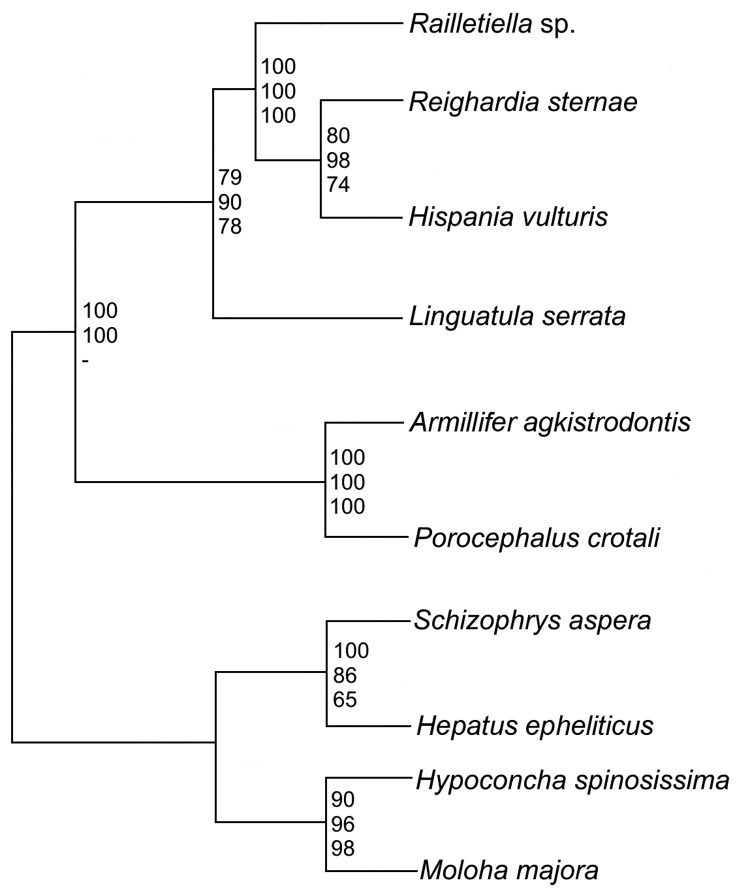
Rectangular cladogram based on 18S rRNA gene sequences of 6 pentastomid species. The tree was rooted with 4 branchiura sequences as an outgroup. The numbers at nodes represent bootstrap values based on 100 replicates (neighbor joining/maximum likelihood/maximum parsimony). Both subgroups of the Pentastomida, the Cephalobaenida (*Reighardia sternae, Hispania vulturis*, and *Raillietiella* spp.) and the Porocephalidae (*Porocephalus crotali* and *Armillifer agkistrodontis*) are well supported. The choice of outgroup had no effect on tree topology but did in some cases reduce bootstrap values.

The 18S rRNA gene of *L. serrata* is 1,834 bp long with a GC content of 49.3%. Sequence similarities to other pentastomids range from 90.0% (*Armillifer agkistrodontis, Porocephalus crotali*) to 90.8% (*Raillietiella* spp.). However, the distance between *L. serrata* and the other species is larger than that between any other species. Cluster analyses resulted in homologous consensus trees independent of the evolutionary model used (Figure 2). Notably, *L. serrata* grouped with the Cephalobaenida, supported by high bootstrap values, although in traditional classification it is assigned to the Porocephalida. To date only 5 other 18S rDNA sequences of pentastomids are available, and the sequence of *P. crotali* is incomplete, limiting the basis for cluster analysis to only 1,560 aligned sites. Even when a cladogram without *P. crotali* and on a basis of 1,780 aligned sites was constructed, *L. serrata* remained in the Cephalobaenida. In fact, at least 1 morphologic feature might support this position: typical Porocephalida, such as those in the genus *Armillifer*, have their hooks horizontally lined up, whereas the hooks of cephalobaenids are pairwise obliquely arranged, as in those in the genus *Linguatula* (*14*).

## Conclusions

This study indicates that rearrangements in the current classification scheme of the Pentastomida might be necessary. We hope that our data will be an impetus for a comprehensive phylogenetic study of this group of parasites.

## References

[1] Siavashi MR, Assmar M, Vatankhah A. Nasopharyngeal pentastomiasis (Halzoun): report of 3 cases. Iran J Mol Sci. 2002;27:191–2.

[2] Yagi H, el Bahari S, Mohamed HA, Ahmed el-R S, Mustafa B, Mahmoud M, . The Marrara syndrome: a hypersensitivity reaction of the upper respiratory tract and buccopharyngeal mucosa to nymphs of *Linguatula serrata.* . Acta Trop. 1996;62:127–34. 10.1016/S0001-706X(96)00017-4 9025980

[3] Tappe D, Büttner DW. Diagnosis of human visceral pentastomiasis. PLoS Negl Trop Dis. 2009;5:e320. 10.1371/journal.pntd.0000320 19238218 PMC2643528

[4] Deweese MW, Murrah WF, Caruthers SB. Case report of a tongue worm (*Linguatula serrata*) in the anterior chamber. Arch Ophthalmol. 1962;68:587–9. 14027430 10.1001/archopht.1962.00960030591004

[5] Hunter WS, Higgins RP. An unusual case of human porocephaliasis. J Parasitol. 1960;48:68–70. 10.2307/3275336

[6] Sousaefaro B, Pinhao RC. An isolated case of ocular parasitosis caused by *Linguatula serrata.* J Soc Cienc Med Lisb. 1964;128:401–20. 14173610

[7] Lang Y, Garzozi H, Epstein Z, Barkay S, Gold D, Lengy J. Intraocular pentastomiasis causing unilateral glaucoma. Br J Ophthalmol. 1987;71:391–5. 10.1136/bjo.71.5.391 3495294 PMC1041175

[8] Lazo RF, Hidalgo E, Lazo JE, Bermeo A, Llaguno M, Murillo J, Ocular linguatuliasis in Ecuador: case report and morphometric study of the larva of *Linguatula serrata.* Am J Trop Med Hyg. 1999;60:405–9. 10466969 10.4269/ajtmh.1999.60.405

[9] Møller OS, Olesen J, Avenant–Oldewage A, Thomsen PF, Glenner H. First maxillae suction discs in branchiura (Crustacea): development and evolution in light of the first molecular phylogeny of branchiura,pentastomida, and other “maxillopoda.”. Arthropod Struct Dev. 2008;37:333–46. 10.1016/j.asd.2007.12.002 18394959

[10] Wingstrand KG. Comparative spermatology of a pentastomid, *Raillietiella hemidactyli*, and a branchiurian crustacean, *Argulus foliaceus*, with a discussion of pentastomid relationships. Kongelige Danske Videnskabemes Selskab Biologiske Skrifter. 1972;19:1–72.

[11] Lavrov DV, Brown WM, Boore JL. Phylogenetic position of the Pentastomida and (pan)crustacean relationships. Proc Biol Sci. 2004;271:537–44. 10.1098/rspb.2003.2631 15129965 PMC1691615

[12] Almeida WO, Christoffersen ML, Amorim DS, Eloy ECC. Morphological support for the phylogenetic positioning of Pentastomida and related fossils. Revista Biotemas. 2008;21:81–90.

[13] Walochnik J, Michel R, Aspöck H. A molecular biological approach to the phylogenetic position of the genus *Hyperamoeba.* J Eukaryot Microbiol. 2004;51:433–40. 10.1111/j.1550-7408.2004.tb00391.x 15352326

[14] Doucet J. Contribution á l’etude anatomique, histologique et histochimique des Pentastomes (Pentastomida). Paris: Office de la recherché scientifique et technique Outre–Mer (O.R.S.T.O.M.); 1965.

[15] Leschnik M, Löwenstein M, Edelhofer R, Kirtz G. Imported non-endemic, arthropod-borne and parasitic infectious diseases in Austrian dogs. Wien Klin Wochenschr. 2008;120:59–62. 10.1007/s00508-008-1077-3 19066775

